# Relationship between ideal cardiovascular health score and perioperative acute kidney injury: A case‐control study

**DOI:** 10.1002/clc.24159

**Published:** 2023-09-19

**Authors:** Jin‐Qiu Li, Yuan An, Tai Wang, Ping‐Xuan Guo, Shuo‐Hua Chen, Bing‐Bing Liu, Hai Liu

**Affiliations:** ^1^ Department of Anesthesiology Kailuan General Hospital Tangshan China; ^2^ Operating Theatre Kailuan General Hospital Tangshan China; ^3^ Department of Cardiology Kailuan General Hospital Tangshan China

**Keywords:** acute kidney injury, cardiovascular health score, surgery

## Abstract

**Background:**

Maintaining ideal cardiovascular health scores (CHS) may indirectly contribute to reducing the risk of perioperative acute kidney injury (AKI), which has never been explored previously. In this study, we aimed to explore the relationship between CHS and AKI and provide new ideas for AKI prevention and treatment.

**Methods:**

We examined the effects of CHS on the occurrence of AKI among 2783 participants from the Kailuan study, who received general anesthesia during noncardiac surgery from 2016 to 2020. The odds ratios (ORs) and 95% confidence intervals (95% CIs) for AKI were calculated by using the logistic regression.

**Results:**

Among 2783 participants 187 were diagnosed with perioperative AKI. We found an inverse relationship between the CHS scores and the risk of AKI. Participants with CHS score ≥ 10 had 57% decreased risk of AKI (OR = 0.43, 95% CI = 0.23, 0.79), compared with participants with CHS score ≤ 7, especially in men (OR = 0.39, 95% CI: 0.20, 0.76). In addition, participants who never smoked, exercised frequently, and had normal blood pressure had decreased risk of AKI, with corresponding ORs (95% CIs) of 0.66 (0.47, 0.91), 0.73 (0.60, 0.92), and 0.46 (0.28, 0.75), respectively.

**Conclusions:**

CHS was strongly associated with the risk of perioperative AKI, and higher CHS scores were associated with a lower risk of AKI. Further research is needed to explore the long‐term effects of achieving and maintaining an ideal CHS on AKI risk.

## Graphical abstract

In the current study, we found that cardiovascular health scores (CHS) score strongly associated with the risk of perioperative acute kidney injury (AKI), with higher CHS scores associated with a lower risk of AKI, These findings provide a theoretical basis and clinical evidence for preventing perioperative AKI.

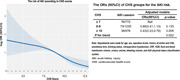

## 1 INTRODUCTION

Perioperative acute kidney injury (AKI) refers to the sudden and rapid loss of kidney function within a few hours or days of a major surgical procedure or anesthesia. It is a common and serious complication of surgery and affects up to 30% of patients who undergo major surgical procedures.^1^, ^2^ The pathogenesis of perioperative AKI is multifactorial and involves decreased renal blood flow, inflammation, oxidative stress, and direct toxicity from drugs and contrast agents.^3^ Several risk factors for perioperative AKI have been identified, including advanced age, pre‐existing chronic kidney disease, diabetes, hypertension, congestive heart failure, liver disease, and prolonged surgery time.^4^ Diagnosis of perioperative AKI is based on the measurement of serum creatinine and urine output, and AKI severity is classified using the Kidney Disease Improving Global Outcomes (KDIGO) criteria.^5^, ^6^ Perioperative AKI management involves the identification and treatment of underlying risk factors and the optimization of hemodynamic parameters, such as blood pressure and fluid balance. Research on perioperative AKI has focused on identifying new biomarkers for early detection, elucidating its pathophysiology, and developing new treatments to prevent or reduce the incidence and severity of AKI.

The ideal cardiovascular health score (CHS) refers to a set of lifestyle choices and health measures that promote optimal cardiovascular health and reduce the risk of cardiovascular diseases. The American Heart Association (AHA) defines seven key health factors and behaviors that contribute to ideal cardiovascular health: smoking, physical activity, body mass index (BMI), diet (salt intake), total cholesterol (TC), blood pressure, and blood glucose.^7^, ^8^ Several cardiovascular risk factors, such as hypertension, diabetes, and obesity, have been identified as independent risk factors for AKI. These risk factors can lead to systemic inflammation, endothelial dysfunction, and impaired renal blood flow, which can increase a patient's susceptibility to AKI during the perioperative period.^9^, ^10^ Practicing ideal cardiovascular health behaviors and maintaining optimal cardiovascular health may indirectly reduce the risk of perioperative AKI.

Although CHS may not directly prevent AKI during the perioperative period, it can contribute to overall cardiovascular and renal health and potentially reduce the risk of AKI and its complications, which has been poorly explored previously. This study aimed to explore the relationship between CHS and AKI and provide new ideas for AKI prevention and treatment.

## 2 METHODS

### 2.1 The Kailuan study and study population

The Kailuan study was a prospective cohort study that was conducted in the Kailuan community, situated in the city of Tangshan. Tangshan, located approximately 150 km southeast of Beijing, is a major center for coal mining in Hebei Province. Kailuan study enrolled 101 510 participants between June 2006 and October 2007 and the cohort included 81 110 men and 20 400 women aged 18–98 years. All subjects underwent follow‐up medical examinations every 2 years. The majority of participants, over 95%, were of Han Chinese ethnicity. Ethical approval for the study protocol was obtained from Kailuan General Hospital. All participants provided written informed consent.^11^

Between 2016 and 2021, 2983 participants in the Kailuan Study received general anesthesia during noncardiac surgery. We excluded 200 participants for whom CHS data were not available, and a total of 2783 participants were included in this study (Supporting Information: Figure S1).

### 2.2 Collection of clinical data and relevant definitions

For details on the epidemiological indicators, anthropometric measurements, and biochemical indicator tests evaluated in this study, please refer to prior literature published by our research team.^12^, ^13^ The general clinical data of the selected individuals were collected through questionnaire surveys, and the information included smoking status, physical exercise, salt intake, any histories of diabetes, hypertension, abnormal blood lipid levels, heart failure, and medication history. Smoking was defined as smoking at least one cigarette/day on average over the past year, and drinking was defined as consuming at least 100 mL of hard liquor (with an alcohol content of 50% or more) per day on average over the past year, continuously, for at least 1 year. Individuals who had quit smoking or drinking for less than 1 year were still considered smokers or drinkers. Regular physical exercise was defined as exercising at least three times a week, with each session lasting at least 30 minutes, while occasional physical exercise was defined as exercising 1–2 times/week, with each session lasting at least 30 minutes. Anthropometric and biochemical indicators of the selected individuals were collected from medical examination records. The BMI was calculated as BMI = weight/height^2^ (kg/m^2^).

Blood pressure in the right brachial artery was measured using a calibrated mercury sphygmomanometer. Systolic blood pressure (SBP) and diastolic blood pressure (DBP) were determined using the first and fifth Korotkoff sounds, respectively. Three consecutive measurements were taken, with 1–2 minutes between each measurement, and the average value was recorded. For biochemical indicator testing, fasting (at least 8 hours) blood samples (5 mL) were collected from the median cubital vein on the day of the medical examination. A Hitachi 7600 biochemical analyzer was used for standardized testing. The main indicators included fasting blood glucose (FBG), TC, triglycerides, C‐reactive protein (CRP), and other measures.

Fluid transfusion volume, blood transfusion volume, urinary volume, bleeding volume, anesthesia time, intraoperative blood pressure, operation levels, and ASA Physical Status Classification System scores were obtained from the intraoperative anesthesia records.

### 2.3 Evaluation of health behavior factors

Seven health behavior factors were evaluated, and each was assigned as ideal, moderate, or poor.^14^ The specific evaluation criteria were as follows: (1) Smoking: no smoking was considered ideal, past smoking, or quitting was considered moderate, and current smoking was considered poor. (2) BMI: <25 kg/m^2^ was ideal, 25–29.9 kg/m^2^ was moderate, and ≥30 kg/m^2^ was poor. (3) Physical exercise: Regular exercise was ideal, occasional exercise was moderate, and no exercise was poor. (4) Healthy diet: Preference for low salt intake (<6 g salt/day) was ideal, approximately 6–10 g salt/day was moderate, and high salt intake (>10 g salt/day) was poor. We also evaluated the following health factors: (1) TC: In the absence of medication, <5.18 mmol/L was ideal, 5.18–6.21 or <5.18 mmol/L with lipid‐lowering medication was moderate, and ≥6.22 or ≥5.18 mmol/L with lipid‐lowering medication was poor. (2) Blood pressure: In the absence of medication, <120/80 mmHg (1 mmHg = 0.133 kPa) was ideal, 120–139/80–89 or <120/80 mmHg with antihypertensive medication was moderate, and ≥140/90 or ≥120/80 mmHg with antihypertensive medication was poor. (3) FBG: In the absence of medication, <5.6 mmol/L was ideal, 5.6–6.9 or <5.6 mmol/L with antidiabetic medication was moderate, and ≥7.0 or ≥ 5.6 mmol/L with antidiabetic medication was poor.

### 2.4 CHS calculation and categorization

Each of the seven cardiovascular health behavior factors was assigned a score of two points for ideal, one point for moderate, and zero points for poor.^15^ The overall CHS value represents the total score of the seven items. Based on CHS, the selected population was divided into three groups: CHS ≤ 7 group, 8 ≤ CHS ≤ 9 group, and CHS ≥ 10 group.

### 2.5 Diagnosis of perioperative AKI

According to current KDIGO guidelines,^6^ AKI is diagnosed based on two functional markers: serum creatinine increase and urine output decrease. Considering serum creatinine levels, AKI presents as a sudden increase in serum creatinine by at least 0.3 mg/dL (26.5 µmol/L) within 48 hours or an increase in serum creatinine to at least 1.5 times the baseline value, which is known or presumed to have occurred within the past 7 days. Considering urine output, AKI presents as decreased urine output, also known as oliguria, which is defined as urine output of less than 0.5 mL/kg/h for at least 6 hours. However, it is important to note that urine output alone may not be a reliable indicator in some individuals, such as in patients taking diuretics or those with pre‐existing kidney disease.

### 2.6 Statistical analysis

Health examination data were imported by trained personnel and uploaded through the network to the computer center at Kailuan General Hospital, into an Oracle 10.2 database. Statistical analyses were performed using SAS software (SAS Institute) version 9.4. Normally distributed continuous data are presented as mean ± standard deviation, and comparisons among multiple groups were analyzed using one‐way analysis of variance. Skewed distributed continuous data are presented as median (interquartile range), and comparisons were conducted using a nonparametric test (Kruskal–Wallis test). Categorical data are presented as frequencies (percentages), and comparisons were performed using the *χ* ^2^ test. Restricted cubic spline curves were used to calculate the continuous form of CHS and the dose–response relationship with the odds ratio (OR) of AKI. Multivariable logistic regression models were used to analyze the influence of the CHS group on the ORs (95% confidence interval [CI]) of AKI, and the ORs of each CHS metric for AKI were further analyzed. Propensity score matching was applied to match the AKI and non‐AKI groups, followed by multivariate logistic regression analysis to reduce the impact of confounding factors on the results. Subgroup analyses on the relationships were also conducted on the variables of age (median, <63 vs. ≥63 years) and sex. Sensitivity analysis was performed by excluding participants who underwent renal surgery, followed by multivariate logistic regression analysis, to minimize the influence of this subgroup on the results. Differences with *p* < .05 (two‐sided) were considered statistically significant.

## 3 RESULTS

### 3.1 General clinical characteristics

Our study included a final population of 2783 participants, with 772, 1035, and 976 individuals in the CHS score ≤ 7 group, 8 ≤ CHS score ≤ 9 group, and CHS score ≥ 10 group, respectively. Age, SBP, DBP, FBG, BMI, TC, CRP, hemoglobin (Hb), fluid transfusion volume, blood transfusion volume, urinary volume, bleeding volume, and anesthesia time differed significantly among the three groups (all *p* < .05; Table 1). The percentages of men, hypertension, diabetes, chronic heart disease (CHD), stroke, intraoperative hypotension, physical activity, salt intake, smoking, alcohol consumption, and ASA Physical Status Classification System differed between the groups (all *p* < .05; Table 1). We further divided the subjects according to whether they had AKI and explored the general clinical information of the two groups (Supporting Information: Table S1).

**Table 1:** Baseline characteristics of the study population stratified by CHS scores.

	CHS scores			*p*‐Value
	≤7	8–9	≥10	
*n*	772	1035	976	
Age (year)	61.87 ± 8.92	62.94 ± 10.05	59.89 ± 12.50	<.001
Male gender (%)	713 (92.36)	845 (81.64)	629 (64.45)	<.001
BMI (kg/m^2^)	27.01 ± 3.33	25.54 ± 3.30	23.69 ± 2.86	<.001
SBP (mmHg)	140.26 ± 19.47	133.19 ± 18.07	118.79 ± 15.51	<.001
DBP (mmHg)	90.85 ± 11.06	85.49 ± 9.96	77.06 ± 9.10	<.001
TC (mmol/L)	5.48 ± 1.25	4.94 ± 1.04	4.52 ± 0.89	<.001
FBG (mmol/L)	6.17 ± 1.87	5.35 ± 1.30	4.95 ± 0.73	<.001
CRP (mg/L)	3.00 (2.05–7.80)	3.00 (1.60–7.20)	3.00 (1.20–6.20)	<.001
Hb (g/L)	143.59 ± 17.08	140.73 ± 16.78	136.98 ± 17.42	<.001
Fluid transfusion volume (per 100 mL)	10.00 (10.00–15.00)	10.00 (10.00–15.00)	10.00 (10.00–15.00)	.001
Blood transfusion volume (mL)	0.00 (0.00–0.00)	0.00 (0.00–0.00)	0.00 (0.00–0.00)	.048
Urinary volume (mL)	150 (0.00–200.00)	150 (150.00–200.00)	150 (0.00–150.00)	<.001
Bleeding volume (mL)	20.00 (5.00–50.00)	20.00 (5.00–50.00)	10.00 (5.00–50.00)	<.001
Anesthesia time (h)	2.10 (1.40–3.10)	2.10 (1.44–3.12)	2.00 (1.30–2.80)	<.001
Hypertension (%)	404 (52.33)	417 (40.29)	207 (21.21)	<.001
Diabetes (%)	206 (26.75)	154 (14.91)	50 (5.14)	<.001
CHD (%)	117 (15.16)	103 (9.95)	67 (6.86)	<.001
Stroke (%)	127 (16.45)	149 (14.40)	67 (6.86)	<.001
Intraoperative hypotension (%)	295 (38.21)	460 (44.44)	369 (37.81)	.004
Physical activity (%)				<.001
Never	193 (25.00)	124 (11.98)	43 (4.41)	
Occasionally	492 (63.73)	746 (72.08)	725 (74.28)	
Frequently	87 (11.27)	165 (15.94)	208 (21.31)	
Perceived salt intake (%)				<.001
Low (<6 g/d)	57 (7.38)	95 (9.18)	135 (13.83)	
Intermediate (6–10 g/d)	538 (69.69)	853 (82.42)	815 (83.50)	
High (>10 g/d)	177 (22.93)	87 (8.41)	26 (2.66)	
Smoking status (%)				<.001
Never	215 (27.85)	603 (58.26)	812 (83.20)	
Past or occasionally	79 (10.23)	152 (14.69)	94 (9.63)	
Frequently	478 (61.92)	280 (27.05)	70 (7.17)	
Drinking status (%)				<.001
Never	500 (64.77)	736 (71.11)	793 (81.25)	
Past or occasionally	26 (3.37)	21 (2.03)	9 (0.92)	
Frequently	246 (31.87)	278 (26.86)	174 (17.83)	
Operation levels (%)				.173
I (II)	12 (1.55)	9 (0.87)	12 (1.23)	
III	190 (24.61)	222 (21.45)	257 (26.33)	
IV	111 (14.38)	163 (15.75)	140 (14.34)	
Unknown	459 (59.46)	641 (61.93)	567 (58.09)	
ASA (%)				<.001
I	29 (3.76)	46 (4.44)	75 (7.68)	
II	578 (74.87)	783 (75.65)	768 (78.69)	
III	144 (18.65)	187 (18.07)	120 (12.30)	
IV (V)	21 (2.72)	19 (1.84)	13 (1.33)	

Abbreviations: ASA, ASA physical status classification system; BMI, body mass index; CHD, chronic heart disease; CHS, cardiovascular health scores; CRP, C‐reactive protein; DBP, diastolic blood pressure; FBG, fasting blood glucose; Hb, hemoglobin; SBP, systolic blood pressure; TC, total cholesterol.

### 3.2 Relationship between CHS levels and AKI

**Figure 1:**
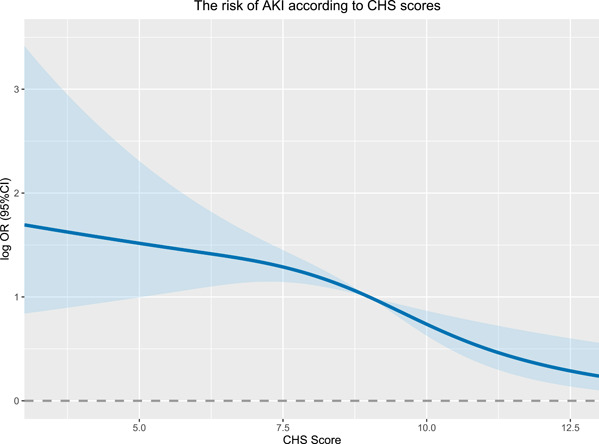
The dose–response relationship between CHS and AKI risk. AKI, acute kidney injury; CHS, cardiovascular health scores; CI, confidence interval; OR, odds ratio.

**Figure 2:**
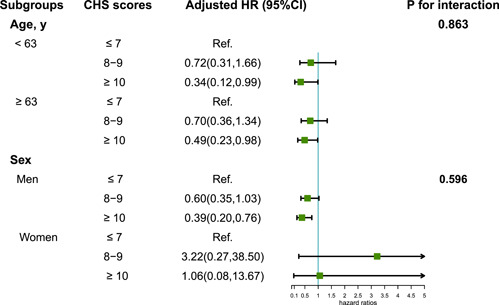
Subgroup analysis of the relationship between CHS and AKI risk. AKI, acute kidney injury; CHS, cardiovascular health scores; CI, confidence interval; HR, hazard ratio.

Among the 2783 participants in the Kailuan study who received general anesthesia during noncardiac surgery from 2016 to 2021. A total of 187 were diagnosed with perioperative AKI. The inverse relationship between the CHS scores and AKI risk is shown in Figure 1. After adjustments were made for age, sex, operation levels, history of stroke and CHD, anesthesia time, drinking status, intraoperative hypotension, CRP, Hb, fluid and blood transfusion volume, urinary volume, bleeding volume, and ASA Physical Status Classification System, participants with CHS score ≥ 10 had 57% decreased ORs of AKI (OR = 0.43, 95% CI = 0.23, 0.79), compared with participants with CHS score ≤ 7 (Table 2). As shown in Figure 2, in the subgroup analysis, subjects with CHS scores ≥ 10 had reduced ORs of AKI compared to those with CHS scores ≤ 7, across age groups (age <63, OR = 0.34, 95% CI: 0.12, 0.99; age ≥63, OR = 0.49, 95% CI: 0.23, 0.98), as did the male population (OR = 0.39, 95% CI: 0.20, 0.76).

**Table 2:** The ORs (95% CI) of CHS groups for the AKI risk.

	AKI cases/*n*	Crude models		Adjusted models	
		ORs (95% CI)	*p*‐Value	ORs (95% CI)	*p*‐Value
CHS					
≤7	76/772	Ref.		Ref.	
8–9	73/1035	0.69 (0.50, 0.97)	.033	0.68 (0.41, 1.13)	.135
≥10	38/976	0.37 (0.25, 0.56)	<.001	0.43 (0.23, 0.79)	.006
*P* for trend			<.001		.022

*Note*: Adjustments were made for age, sex, operation levels, history of stroke and chronic heart disease, anesthesia time, drinking status, intraoperative hypotension, C‐reactive protein, HGB, fluid and blood transfusion volume, urinary volume, bleeding volume, and ASA physical status classification system.

Abbreviations: AKI, acute kidney injury; CHS, cardiovascular health scores; CI, confidence interval; HGB, hemoglobin; OR, odds ratio.

### 3.3 Sensitivity analysis and CHS components

In the sensitivity analysis, participants who underwent kidney surgery were excluded to eliminate the possible effects of kidney surgery on the primary outcome. We further used propensity score matching to adjust for imbalances in covariates between patients with AKI and the non‐AKI population, and similar results were obtained for the primary findings (Supporting Information: Tables S2 and S3). We further analyzed the relationship between each CHS component and AKI risk (Table 3). After adjusting for age, sex, operation level, history of stroke and CHD, anesthesia time, drinking status, intraoperative hypotension, CRP, Hb, fluid and blood transfusion volume, urinary volume, bleeding volume, ASA Physical Status Classification System, and all CHS components, participants who never smoked, exercised frequently, and had normal blood pressure had decreased risk of AKI, with corresponding ORs (95% CI) of 0.66 (0.47, 0.91), 0.73 (0.60, 0.92), and 0.46 (0.28, 0.75), respectively.

**Table 3:** The ORs (95% CI) of each CHS component for the AKI risk.

CHS components	Crude models		Mutually adjusted models	
	ORs (95% CI)	*p*‐Value	ORs (95% CI)	*p*‐Value
Smoking status				
Frequently	Ref.		Ref.	
Past or occasionally	0.74 (0.45, 1.22)	.239	0.76 (0.46, 1.27)	.300
Never	**0.66 (0.48, 0.91)**	**.010**	**0.66 (0.47, 0.91)**	**.012**
BMI (kg/m^2^)				
≥30	Ref.		Ref.	
25–29.9	0.72 (0.43, 1.18)	.190	0.74 (0.44, 1.22)	.234
<25	0.62 (0.37, 1.02)	.062	0.72 (0.43, 1.21)	0.213
Physical exercise				
Never	Ref.		Ref.	
Occasionally	0.96 (0.62, 1.48)	.842	1.09 (0.69, 1.70)	.723
Frequently	**0.70 (0.58, 0.90)**	**.001**	**0.73 (0.60, 0.92)**	**.003**
Salt intake (g/d)				
High (>10)	Ref.		Ref.	
Intermediate (6–10)	0.95 (0.58, 1.54)	.822	1.05 (0.64, 1.74)	.846
Low (<6)	1.18 (0.63, 2.20)	.601	1.25 (0.66, 2.34)	.493
TC (mmol/L)				
≥6.22	Ref.		Ref.	
5.18‐6.21	0.80 (0.49, 1.31)	.375	0.88 (0.53, 1.47)	.633
<5.18	0.69 (0.44, 1.10)	.120	0.83 (0.52, 1.34)	.453
Blood pressure (mmHg)				
≥140/90	Ref.		Ref.	
120–139/80–89	0.58 (0.42, 0.80)	<.001	0.60 (0.43, 0.84)	.003
<120/80	**0.42 (0.26, 0.68)**	**<.001**	**0.46 (0.28, 0.75)**	**.002**
FBG (mmol/L)				
≥7	Ref.		Ref.	
5.6–6.9	0.94 (0.54, 1.63)	.823	1.00 (0.57, 1.75)	.999
<5.6	0.69 (0.42, 1.14)	.149	0.82 (0.49, 1.37)	.443

*Note*: Results presented with bold valued were statistically significant with all *p* value < 0.05. Adjustments were made for age, sex, operation levels, history of stroke and chronic heart disease, anesthesia time, drinking status, intraoperative hypotension, C‐reactive protein, HGB, fluid and blood transfusion volume, urinary volume, bleeding volume, ASA physical status classification system, and all cardiovascular health score components.

Abbreviations: AKI, acute kidney injury; BMI, body mass index; CHS, cardiovascular health scores; CI, confidence interval; FBG, fasting blood glucose; HGB, hemoglobin; OR, odds ratio; TC, total cholesterol.

## 4 DISCUSSION

The identification of modifiable risk factors is crucial for AKI prevention and management. In the current study, we found that CHS scores are strongly associated with the risk of perioperative AKI, with higher CHS scores associated with a lower risk of AKI, especially in men. In addition, the CHS components, including nonsmoking status, exercise, and normal blood pressure, were associated with a lower risk of AKI. These findings provide a theoretical basis and clinical evidence for preventing perioperative AKI.

The CHS score is a composite measure of cardiovascular health that is based on several modifiable risk factors, which include smoking status, BMI, physical activity, diet, blood pressure, blood glucose, and cholesterol levels. While few studies have investigated the association between CHS and AKI, numerous studies have consistently shown that individuals with a higher CHS have a significantly lower risk of several health outcomes, including cardiovascular disease^16^, ^17^ (coronary artery disease, stroke, and heart failure), all‐cause mortality,^18^ metabolic health^19^ (obesity, type 2 diabetes, and metabolic syndrome), and cognitive function.^20^ In 2010, the AHA established ambitious goals to enhance the cardiovascular health of the population by 20% and reduce mortality from cardiovascular diseases and stroke by 20% by the year 2020.^21^ Achieving these goals necessitates the development of new strategic approaches to cardiovascular health promotion and disease prevention using the AHA's research, clinical results, public health policies, and patient advocacy initiatives for the coming decade and beyond.

In our study, we investigated the association between each CHS component and the occurrence of perioperative AKI. Our findings revealed that nonsmoking status, regular physical activity, and optimal blood pressure were significantly associated with a reduced risk of developing AKI during the perioperative period. Notably, smoking has previously been identified as a risk factor for AKI.^22^ Harmful chemicals present in tobacco smoke can lead to endothelial dysfunction and inflammation, which affects the blood vessels and impairs kidney function.^23^ Smoking also causes vasoconstriction, reduces renal blood flow, and promotes blood clot formation, which can contribute to kidney damage.^24^ Additionally, smoking has been linked to increased oxidative stress and the release of inflammatory markers that further exacerbate kidney injury.^25^ The relationship between blood pressure and AKI can be explained by several mechanisms, including impaired renal blood flow, increased glomerular pressure, enhanced oxidative stress, and inflammation.^26^ Notably, controlling blood pressure through lifestyle modifications and, if necessary, medication is crucial for reducing the risk of AKI. By maintaining blood pressure within the recommended range, individuals can preserve renal blood flow, protect glomerular filtration barrier integrity, and minimize AKI risk.

Maintaining an ideal CHS can potentially decrease the risk of perioperative AKI through several mechanisms, as follows. First, maintaining an ideal CHS provides a healthy cardiovascular system,^27^ which ensures optimal blood flow to all organs, including the kidneys. Good blood flow helps maintain an adequate oxygen and nutrient supply to the kidneys and reduces the risk of kidney injury. Conversely, individuals with compromised cardiovascular health may have reduced blood flow to the kidneys, making them more susceptible to AKI. Second, maintaining an ideal CHS lowers the risk of ischemia.^28^ Ischemia refers to an inadequate blood supply to tissues or organs. During surgery, there can be instances of reduced blood flow to the kidneys due to various factors, such as changes in blood pressure or the use of certain medications. Having an ideal CHS, which includes factors such as blood pressure control and regular exercise, can minimize the risk of ischemia‐related kidney injury. Third, maintaining an ideal CHS reduces inflammation.^29^ Surgery triggers an inflammatory response in the body that contributes to kidney injury. Furthermore, a healthy cardiovascular system is associated with low systemic inflammation. By adhering to a healthy lifestyle, including a balanced diet and regular exercise, individuals can reduce chronic inflammation and potentially mitigate inflammatory responses during surgery, thereby lowering the risk of AKI. Fourth, an ideal CHS minimizes nephrotoxic exposure. Certain medications and substances used during surgery, such as contrast agents and anesthetics, can have nephrotoxic effects, potentially leading to AKI. However, individuals with ideal cardiovascular health may have fewer comorbidities and a reduced need for certain medications, which minimizes their exposure to nephrotoxic substances and consequently decreases their risk of AKI. Fifth, maintaining an ideal CHS allows optimal fluid balance, which is crucial for kidney health. Patients with ideal cardiovascular health are more likely to have a well‐regulated fluid status, which can help prevent fluid overload or dehydration, both of which can contribute to kidney injury during surgery. Overall, perioperative AKI is a significant clinical problem with a complex pathogenesis and multiple risk factors. Further research is required to improve our understanding of the underlying mechanisms, develop more effective preventive strategies, and identify new treatments for this serious complication.

This study represents a pioneering analysis of the association between CHS and AKI, and our findings shed new light on the prevention and management of perioperative AKI. The notable strengths of this study include substantial sample size, the inclusion of numerous relevant covariates, and the application of multiple statistical methods, including propensity scores. It is also important to acknowledge the limitations of this study. First, the study did not incorporate several novel markers of kidney injury, for example, neutrophil gelatinase‐associated lipocalin,^30^ which could have provided additional insights into the relationship between CHS and AKI. These markers may include urinary biomarkers or imaging techniques that can accurately assess renal function and structural changes. Second, it is important to acknowledge that the Kailuan community primarily consists of industrial laborers, which may lead to an imbalance in the distribution of sexes within the study population. However, it is noteworthy that the researchers conducted separate statistical analyses for both sexes, which helped to mitigate the potential impact of this imbalance on the study results. Third, we only used CHS data from a single measurement. CHS may change over time, which may have led to misgrouping of the population. Future studies should focus on long‐term CHS and the impact of changes on health outcomes.

## 5 CONCLUSIONS

The results of this study suggest that CHS is strongly associated with the risk of perioperative AKI, with higher CHS scores being associated with a lower risk of AKI, especially in men. Promoting and maintaining ideal cardiovascular health, as measured by the CHS, should be a public health priority. Strategies that aim to promote cardiovascular health should focus on lifestyle modifications, risk factor management, and promoting an awareness of CHS metrics. Further research is required to explore the long‐term effects of achieving and maintaining an ideal cardiovascular health status on various health outcomes.

## CONFLICT OF INTEREST STATEMENT

The authors declare no conflict of interest.

## Supporting information

Supporting information.

## Data Availability

Data will be made available upon reasonable request.
