# Supplementary material for: Relationship between ideal cardiovascular health score and perioperative acute kidney injury: A case‐control study
Source: Clin Cardiol. 2023 Sep 19;47(1):e24159. doi: 10.1002/clc.24159 (PMC10766002; doi:10.1002/clc.24159)
Supplement: Supplementary file 1 — Supporting information. [file CLC-47-e24159-s001.docx]

**Tables**

**Supplementary Table 1. Baseline characteristics of the study population stratified by AKI status.**

|  | **Non-AKI** | **AKI** | **p-value** |
| --- | --- | --- | --- |
| **n** | 2596 | 187 |  |
| **Age (year)** | 61.34±10.85 | 64.81±9.06 | <0.001 |
| **Male gender (%)** | 2030(78.20) | 157(83.96) | 0.064 |
| **BMI (Kg/m^2^)** | 25.27±3.42 | 25.77±3.46 | <0.001 |
| **SBP (mmHg)** | 129.65±19.51 | 136.35±21.10 | <0.001 |
| **DBP (mmHg)** | 83.74±11.31 | 87.91±12.31 | <0.001 |
| **TC (mmol/L)** | 4.94±1.11 | 5.05±1.22 | <0.001 |
| **FBG (mmol/L)** | 5.42±1.40 | 5.68±1.73 | <0.001 |
| **HGB (g/L)** | 140.30±17.15 | 138.99±18.99 | <0.001 |
| **CRP (mg/L)** | 3.00(1.50-6.90) | 3.00(1.60-7.20) | <0.001 |
| **Fluid transfusion volume (per 100mL)** | 10.00(10.00-15.00) | 15.00(10.00-20.00) | <0.001 |
| **Blood transfusion volume (mL)** | 0.00(0.00-0.00) | 0.00(0.00-280.00) | <0.001 |
| **Urinary volume (mL)** | 150(50-200) | 150(150 -300) | <0.001 |
| **Bleeding volume (mL)** | 20.00(5.00-50.00) | 50.00(10.00-200.00) | <0.001 |
| **Anesthesia time (h)** | 2.00(1.34-3.00) | 2.84(1.84-4.00) | <0.001 |
| **Hypertension (%)** | 928(35.75) | 100(53.48) | <0.001 |
| **Diabetes (%)** | 373(14.41) | 37(19.79) | 0.046 |
| **CHD (%)** | 257(9.90) | 30(16.04) | 0.008 |
| **Stroke (%)** | 310(11.94) | 33(17.65) | 0.022 |
| **Intraoperative hypotension (%)** | 1023(39.41) | 101(54.01) | <0.001 |
| **Physical activity (%)** |  |  | 0.474 |
| **Never** | 334(12.87) | 26(13.90) |  |
| **Occasionally** | 1827(70.38) | 136(72.73) |  |
| **Frequently** | 435(16.76) | 25(13.37) |  |
| **Perceived salt intake (%)** |  |  | 0.634 |
| **Low (<6 g/d)** | 270(10.40) | 20(10.70) |  |
| **Intermediate (6-10 g/d)** | 2062(79.43) | 144(77.01) |  |
| **High (>10 g/d)** | 264(10.17) | 23(12.30) |  |
| **Smoking status (%)** |  |  | 0.036 |
| **Never** | 757(29.16) | 71(37.97) |  |
| **Past or occasionally** | 304(11.71) | 21(11.23) |  |
| **Frequently** | 1535(59.13) | 95(50.50) |  |
| **Drinking status (%)** |  |  | 0.798 |
| **Never** | 1894(72.96) | 135(72.19) |  |
| **Past or occasionally** | 651(25.08) | 47(25.13) |  |
| **Frequently** | 51(1.96) | 5(2.67) |  |
| **Operation levels (%)** |  |  | <0.001 |
| **Ⅰ (Ⅱ)** | 33(1.27) | 0(0) |  |
| **Ⅲ** | 637(24.54) | 32(17.11) |  |
| **Ⅳ** | 365(14.06) | 49(26.20) |  |
| **Unknown** | 1561(60.13) | 106(56.68) |  |
| **ASA (%)** |  |  | <0.001 |
| **Ⅰ** | 145(5.59) | 5(2.67) |  |
| **Ⅱ** | 2005(77.23) | 124(66.31) |  |
| **Ⅲ** | 410(15.79) | 41(21.93) |  |
| **Ⅳ(Ⅴ)** | 36(1.39) | 17(9.09) |  |

**Note: BMI, body mass index; SBP, systolic blood pressure; DBP, diastolic blood pressure; TC, total cholesterol; FBG: fasting blood glucose; CRP: C-reactive protein; Hb, hemoglobin; CHD: chronic heart disease; ASA: ASA physical status classification system.**

**Supplementary Table 2. Baseline characteristics of the study population stratified by AKI status using propensity score matching.**

|  | **Non-AKI** | **AKI** | **p-value** |
| --- | --- | --- | --- |
| **n** | 187 | 187 |  |
| **Age (year)** | 65.44±9.56 | 64.82±9.10 | 0.521 |
| **Male gender (%)** | 159(85.03) | 157(83.96) | 0.775 |
| **BMI (Kg/m^2^)** | 25.77±3.23 | 25.74±3.46 | 0.939 |
| **SBP (mmHg)** | 133.55±18.31 | 136.35±21.10 | 0.134 |
| **DBP (mmHg)** | 86.47±11.04 | 87.91±12.31 | 0.235 |
| **TC (mmol/L)** | 5.12±1.13 | 5.05±1.22 | 0.486 |
| **FBG (mmol/L)** | 5.96±1.97 | 5.68±1.73 | 0.150 |
| **HGB (g/L)** | 138.05±19.39 | 138.99±18.99 | 0.634 |
| **CRP (mg/L)** | 3.00(1.90-8.20) | 3.00(1.60-7.20) | 0.082 |
| **Fluid transfusion volume (per 100mL)** | 15.00(10.00-20.00) | 15.00(10.00-20.00) | 0.578 |
| **Blood transfusion volume (mL)** | 0.00(0.00-0.00) | 0.00(0.00-280.00) | 0.879 |
| **Urinary volume (mL)** | 150(150-300) | 150(150 -300) | 0.812 |
| **Bleeding volume (mL)** | 20.00(5.00-100.00) | 50.00(10.00-200.00) | 0.044 |
| **Anesthesia time (h)** | 2.20(1.54-3.30) | 2.84(1.84-4.00) | 0.023 |
| **Hypertension (%)** | 928(35.75) | 100(53.48) | <0.001 |
| **Diabetes (%)** | 373(14.41) | 37(19.79) | 0.046 |
| **CHD (%)** | 257(9.90) | 30(16.04) | 0.008 |
| **Stroke (%)** | 310(11.94) | 33(17.65) | 0.022 |
| **Intraoperative hypotension (%)** | 1023(39.41) | 101(54.01) | <0.001 |
| **Physical activity (%)** |  |  | 0.289 |
| **Never** | 29(15.51) | 26(13.90) |  |
| **Occasionally** | 123(65.78) | 136(72.73) |  |
| **Frequently** | 25(18.72) | 25(13.37) |  |
| **Perceived salt intake (%)** |  |  | 0.012 |
| **Low (<6 g/d)** | 32(17.11) | 20(10.70) |  |
| **Intermediate (6-10 g/d)** | 146(78.07) | 144(77.01) |  |
| **High (>10 g/d)** | 9(4.81) | 23(12.30) |  |
| **Smoking status (%)** |  |  | 0.640 |
| **Never** | 70(37.43) | 71(37.97) |  |
| **Past or occasionally** | 27(14.44) | 21(11.23) |  |
| **Frequently** | 90(48.13) | 95(50.50) |  |
| **Drinking status (%)** |  |  | 0.440 |
| **Never** | 142(75.94) | 135(72.19) |  |
| **Past or occasionally** | 43(22.99) | 47(25.13) |  |
| **Frequently** | 2(1.07) | 5(2.67) |  |
| **Operation levels (%)** |  |  | 0.359 |
| **Ⅰ (Ⅱ)** | 1(0.53) | 0(0) |  |
| **Ⅲ** | 32(17.11) | 32(17.11) |  |
| **Ⅳ** | 37(19.79) | 49(26.20) |  |
| **Unknown** | 117(62.57) | 106(56.68) |  |
| **ASA (%)** |  |  | 0.195 |
| **Ⅰ** | 7(3.74) | 5(2.67) |  |
| **Ⅱ** | 121(64.71) | 124(66.31) |  |
| **Ⅲ** | 51(27.27) | 41(21.93) |  |
| **Ⅳ(Ⅴ)** | 8(4.28) | 17(9.09) |  |

**Note: BMI, body mass index; SBP, systolic blood pressure; DBP, diastolic blood pressure; TC, total cholesterol; FBG: fasting blood glucose; CRP: C-reactive protein; Hb, hemoglobin; CHD: chronic heart disease; ASA: ASA physical status classification system.**

**Supplementary Table 3. Sensitivity analysis for the relationship between CHS scores and AKI risk.**

|  | **AKI cases/n** | **Crude models** | |  | **Adjusted models** | |
| --- | --- | --- | --- | --- | --- | --- |
| **CHS** |  | **ORs(95%CI)** | ***p*-value** |  | **ORs(95%CI)** | ***p*-value** |
| **Excluding participants received renal surgery** | | | |  |  |  |
| **≤ 7** | 58/685 | Ref. |  |  | Ref. |  |
| **8-9** | 59/941 | 0.72(0.50,1.05) | 0.090 |  | 0.77(0.43,1.35) | 0.360 |
| **≥ 10** | 30/905 | 0.37(0.24,0.58) | <0.001 |  | 0.48(0.25,0.96) | 0.037 |
| ***P* for trend** |  |  | <0.001 |  |  | 0.113 |
| **Using the propensity score matching method** | | | |  |  |  |
| **≤ 7** | 76/89 | Ref. |  |  | Ref. |  |
| **8-9** | 73/65 | 0.96(0.69,2.00) | 0.613 |  | 0.99(0.71,2.13) | 0.577 |
| **≥ 10** | 38/33 | 0.50(0.33,0.89) | 0.002 |  | 0.51(0.34,0.92) | 0.011 |
| ***P* for trend** |  |  | 0.004 |  |  | 0.044 |

**Note: Adjustments were made for age, sex, operation levels, history of stroke and CHD, anesthesia time, drinking status, intraoperative hypotension, CRP, HGB, fluid and blood transfusion volume, urinary volume, bleeding volume, ASA physical status classification system, and all CHS components.**

**
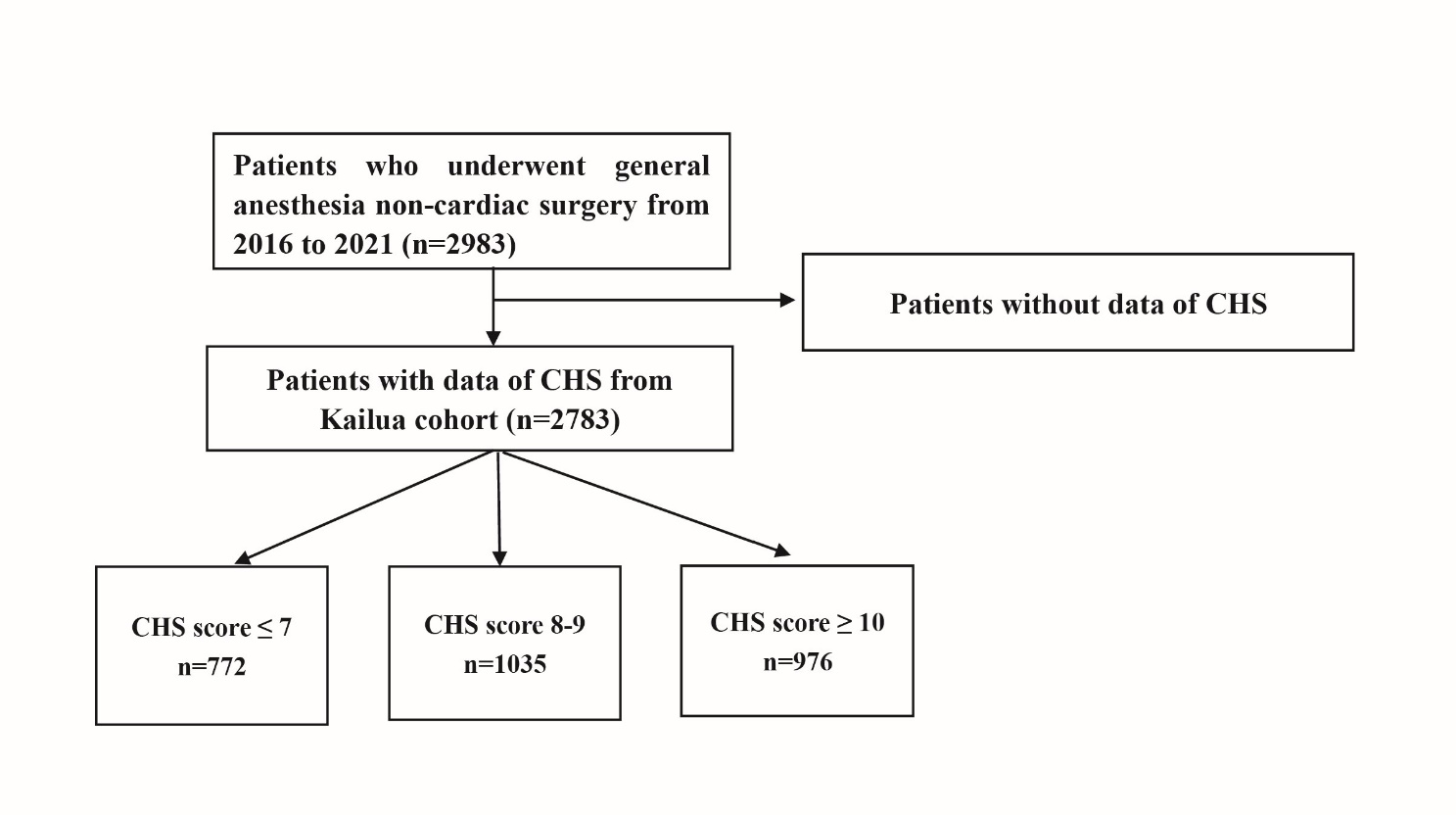
 Supplementary Figure 1. Flow chart of study participants.**
